# Supplementary material for: Identification and functional analysis of non-coding regulatory small RNA FenSr3 in Bacillus amyloliquefaciens LPB-18
Source: PeerJ. 2023 May 15;11:e15236. doi: 10.7717/peerj.15236 (PMC10194069; doi:10.7717/peerj.15236)
Supplement: Supplemental Information 4 [file peerj-11-15236-s004.zip › KO/CK-vs-T1_map/map04260.html]

KEGG PATHWAY: Cardiac muscle contraction - Reference pathway


|  |  |
| --- | --- |
| **Cardiac muscle contraction - Reference pathway** |  |

[
Pathway menu
| Organism menu
| Pathway entry
| Show description
| User data mapping
]

Contraction of the heart is a complex process initiated by the electrical excitation of cardiac myocytes (excitation-contraction coupling, ECC). In cardiac myocytes, Ca2+ influx induced by activation of voltage-dependent L-type Ca channels (DHP receptors) upon membrane depolarization triggers the release of Ca2+ via Ca2+ release channels (ryanodine receptors) of sarcoplasmic reticulum (SR) through a Ca2+ -induced Ca release (CICR) mechanism. Ca2+ ions released via the CICR mechanism diffuse through the cytosolic space to contractile proteins to bind to troponinC resulting in the release of inhibition induced by troponinI. The Ca2+ binding to troponinC thereby triggers the sliding of thin and thick filaments, that is, the activation of a crossbridge and subsequent cardiac force development and/or cell shortening. Recovery occurs as Ca2+ is pumped out of the cell by the Na+/Ca2+ exchanger (NCX) or is returned to the sarcoplasmic reticulum (SR) by sarco(endo)plasmic Ca2+ -ATPase (SERCA) pumps on the non-junctional region of the SR.

|  |  |  |
| --- | --- | --- |
| Reference pathway | 184% 150% 122% 100% 82% 67% 55% | 图片下载 |
